# ‘At Risk Mental State’: An Audit of Tier 3 Clinical Standards

**DOI:** 10.1192/bjo.2025.10694

**Published:** 2025-06-20

**Authors:** Adisha Kapila, Megan Viegas, Jonathan Bradley

**Affiliations:** South West London and St George’s Mental Health NHS Trust, London, United Kingdom

## Abstract

**Aims:** The At-Risk Mental State (ARMS), an attenuated psychotic syndrome, represents a critical period of vulnerability for the development of psychosis. Early identification and evidence-based intervention are crucial to reducing distress, improving long-term outcomes and public health costs. There are clear recommendations stated by National Institute for Health and Care Excellence (NICE) for the optimal management of ARMS in children and young people including early identification, access to psychological therapy and care co-ordination. Baseline audit data collected from Tier 3 teams within South West London and St George’s NHS Mental Health Trust (SWLSTG) highlighted significant variation in clinicians’ confidence and knowledge about ARMS, notably its identification criteria and optimal management. This audit sought to enhance clinician expertise of “At Risk Mental State” (ARMS) within Tier 3 Child and Adolescent Mental Health Services (CAMHS).

**Methods:** An educational intervention was developed to address the identified knowledge gaps. This included a 30-minute didactic teaching seminar covering ARMS diagnostic criteria, clinical challenges, and management guidelines, delivered during the CPD slot for four multidisciplinary teams across SWLSTG. Key topics included the Comprehensive Assessment of At-Risk Mental States (CAARMS), the role of psychological and family interventions, and current NHS England guidelines that included discouraging antipsychotic use in ARMS management.

**Results:** Post-intervention analysis showed improved clinician confidence in both ARMS identification and management. However, all participants indicated a need for additional support. Proposed ideas included specialist training (e.g. CBT for Psychosis and Family Interventions for Psychosis), access to validated assessment tools, appropriate funding for care co-ordination and/or the establishment of a dedicated ARMS service. Qualitative feedback also emphasised the diagnostic difficulty in this population and sociodemographic bias when identifying ARMS within CAMHS settings, highlighting the need for a public health approach to prevention of psychosis.

**Conclusion:** This project illustrates the effectiveness of a simple targeted educational initiative in improving ARMS-related competencies among Tier 3 CAMHS clinicians. It also highlights the importance of integrating structured tools and specialised pathways to optimise care for individuals at high risk of psychosis. Our next steps are to consider strategies to improve the standard of care provided for young people with ARMS. This includes further psychoeducation resources and a funding application for specialist training for Tier 3 psychologists.

